# Supplementary material for: Differences in soil carbon fractions and microbial communities and their underlying mechanisms between assisted natural regeneration and plantation forests in subtropical China
Source: PLoS One. 2026 Jan 8;21(1):e0339350. doi: 10.1371/journal.pone.0339350 (PMC12782447; doi:10.1371/journal.pone.0339350)
Supplement: Attachment — Submitted filename: 4.Response to Reviewers.docx [file pone.0339350.s004.docx]

### ****Response to Editor****

We sincerely appreciate the editor’s careful review and constructive suggestions regarding the manuscript format and structure. We have thoroughly addressed all the editorial comments, and the specific modifications are summarized below:

**1.Please ensure that your manuscript meets PLOS ONE's style requirements, including those for file naming.**

Thank you for your valuable feedback regarding the style requirements. I have carefully reviewed and revised the manuscript to ensure that it fully complies with the PLOS ONE formatting guidelines, including the appropriate file naming conventions.

The manuscript has been updated according to the provided templates, and I have made sure all formatting elements are in alignment with the journal's specifications. The files have been properly renamed in accordance with the submission requirements.

Please let me know if any further adjustments are needed.

**2. In your Methods section, please provide additional information regarding the permits you obtained for the work. Please ensure you have included the full name of the authority that approved the field site access and, if no permits were required, a brief statement explaining why.**

Thank you for your suggestion. We confirm that the study was conducted at Chenda Forest Farm, which is part of China’s National Forest Ecosystem Research Station network. The site is managed for long-term ecological research and public scientific use. Therefore, no special permits were required for soil sampling.

We have now added a corresponding statement in the Materials and Methods section to clarify the research site authorization and compliance status.

**3. Please state what role the funders took in the study. Please include this amended Role of Funder statement in your cover letter; we will change the online submission form on your behalf.**

Thank you for your comment regarding the role of the funders. I would like to clarify that the funders only provided financial support for the study and had no role in the design of the study, data collection and analysis, decision to publish, or preparation of the manuscript.

I have included this amended statement in the cover letter, and I will ensure that the online submission form is updated accordingly.

This work was supported by the National Natural Science Foundation of China (grant numbers 32192433, 32101495, and 31930071).

Role of the Funder Statement:

The funders had no role in study design, data collection and analysis, decision to publish, or preparation of the manuscript.

**4. Please remove any funding-related text from the manuscript and let us know how you would like to update your Funding Statement. Please include your amended statements within your cover letter; we will change the online submission form on your behalf.**

As requested, we have removed all funding-related text from the manuscript.

Please update the Funding Statement as follows:

[This work was supported by the National Nature Science Foundation of China (NO. 32192433, 32101495 and 31930071).]

Thank you for your assistance in updating the online submission record.

**5. Please confirm at this time whether or not your submission contains all raw data required to replicate the results of your study. Authors must share the “minimal data set” for their submission. PLOS defines the minimal data set to consist of the data required to replicate all study findings reported in the article, as well as related metadata and methods. If your submission does not contain these data, please either upload them as Supporting Information files or deposit them to a stable, public repository and provide us with the relevant URLs, DOIs, or accession numbers. If there are ethical or legal restrictions on sharing a de-identified data set, please explain them in detail (e.g., data contain potentially sensitive information, data are owned by a third-party organization, etc.) and who has imposed them (e.g., an ethics committee). Please also provide contact information for a data access committee, ethics committee, or other institutional body to which data requests may be sent. If data are owned by a third party, please indicate how others may request data access.**

Thank you for your comment regarding the data availability. I would like to confirm that my submission includes the necessary raw data to replicate the study's results. All relevant data, including the minimal data set required by PLOS ONE, have been provided as Supporting Information files with the manuscript.

**6.We note that Figure 1 in your submission contain [map/satellite] images which may be copyrighted. We require you to either (1) present written permission from the copyright holder to publish these figures specifically under the CC BY 4.0 license, or (2) remove the figures from your submission:a. You may seek permission from the original copyright holder of Figure 1 to publish the content specifically under the CC BY 4.0 license.**

Thank you very much for your patience and careful confirmation of the insertion of Figure 1 of my manuscript. In order to fully comply with the journal's copyright policy and CC BY 4.0 licensing requirements, we have adjusted the original image and further optimized the annotations.

First, we want to make it clear that the map image in updated Figure 1 is not derived from Google Maps or any of its associated services.

Specifically:

(1) The world map in panel (a) was generated using QGIS v3.28, with base layers sourced from Natural Earth (https://www.naturalearthdata.com/), which provides free public domain cartographic data.

(2) The elevation map in panel (b) was produced using SRTM DEM data (NASA Shuttle Radar Topography Mission, freely available at https://earthexplorer.usgs.gov/) and visualized in QGIS.

(3) All administrative boundaries were created using shapefiles from GADM database (https://gadm.org/).

We confirm that the manuscript now adheres to the journal’s formatting guidelines. Thank you for your time and consideration. If further clarification or additional information is needed, please feel free to let me know.

**Response to Reviewer1:**

**1) this work did not directly address carbon storage, and the term "C storage" in the title should be replaced with "C fractions" to more accurately reflect the study's focus.**

Thank you for your comment regarding the title of the manuscript. As per your suggestion, I have replaced the term "C storage" with "C fractions" to more accurately reflect the study's focus. The title has been updated accordingly.

2) **INTRODUCTION. The introduction lacks sufficient focus on the major topic. More attention should be given to building a strong theoretical basis for the hypotheses, which are currently missing. Clear articulation of hypotheses is essential. Some extraneous information should be removed, such as L80–85 (discussion on rhizosphere and plant and microbial diversity), as they are not directly relevant to your study. The background on ANR (L41–49) is overly detailed and should be condensed to improve clarity and flow.**

We sincerely appreciate the reviewers' valuable comments on the introduction section. We have carefully revised the manuscript to improve focus, strengthen theoretical foundations, and enhance clarity. The specific modifications are as follows:

We believe these revisions have significantly improved the focus, coherence, and rigor of the Introduction. Thank you for your valuable suggestions.

（1）Clear articulation of hypotheses

We have simplified the final paragraph and explicitly stated the research hypotheses: “[To address these questions, we investigated four forest types in a typical subtropical region of southern China: a secondary natural forest, an ANR forest, a *Castanopsis carlesii* monoculture, and a *Cunninghamia lanceolata* monoculture. Specifically, we aimed to: (1) compare SOC fractions and microbial community composition across different forest management types; and (2) quantify the biotic (microbial traits) and abiotic (soil properties) pathways that drive LC and RC accumulation using structural equation modeling (SEM). This study provides new insights into the dual regulatory mechanisms of SOC stability and supports ANR as a viable strategy for carbon conservation and sustainable forest management. We hypothesize that: (1) assisted natural regeneration (ANR) enhances soil carbon storage by maintaining higher labile carbon fractions (LC) and supporting microbial community stability; and (2) monoculture plantations, especially *Cunninghamia lanceolata*, reduce SOC through losses in LC and shifts in microbial structure toward oligotrophic groups due to increased disturbance and substrate limitation.]”

（2）Strengthening theoretical connections:

The original text “[Soil Organic Carbon (SOC) is a heterogeneous mixture composed of a series of simple and complex molecules, which can be divided into labile and recalcitrant carbon fractions (LC and RC) according to their turnover time.]” has been revised to “[Recent studies have highlighted the importance of differentiating SOC into functional fractions, such as labile carbon (LC) and recalcitrant carbon (RC), to better understand the stability and dynamics of soil carbon pools[12-13].]”

A transitional sentence“[Since SOC fraction dynamics are ultimately governed by microbial-mediated processes, understanding how management-induced environmental filters shape microbial functional guilds is critical to deciphering carbon stabilization mechanisms[18-19].]” has been added before the "Changes in carbon input and soil nutrient availability under different management measures can affect the composition of microbial communities..." paragraph to improve logical flow.

（3）Streamlining background and removing redundant information:

The redundant discussion on “Microorganisms are important components…structure from eutrophic to oligotrophic [28].”，has been deleted and replaced with: “[Changes in carbon input and soil nutrient availability under different management measures can affect the composition of microbial communities, especially the balance between bacteria and fungi, and thus affect the decomposition and stability of SOC components[20].]”

The original text “[Although the proportion of LC in SOC is relatively small, it profoundly affects soil nutrient turnover, C/N/P cycling and organic matter decomposition due to its short residence time and fast cycling. Therefore, it serves as a sensitive indicator of environmental changes caused by forest management practices. In contrast, RC is difficult to be utilized by microorganisms, has a long decomposition period, and plays an important role in determining the long-term stability of soil carbon pool, contributing to soil function and health[9]. Hence, assessing the relative proportion changes of LC and RC may provide more information about the soil carbon pool response to forest management practices than SOC concentration, and is beneficial for achieving better resource and environmental management[10]. The complex process of soil carbon components accumulation, decomposition, and dynamic changes involves multiple mechanisms[11-12].]”has been revised to “[LC, derived from microbial biomass, root exudates, and partially decomposed litter, is sensitive to management-induced disturbance and readily responds to microbial activity[14]. In contrast, RC, which includes chemically complex compounds such as lignin and cellulose, is primarily stabilized through physical protection and mineral association[15].]”

（4）More precise condensation of ANR background (Lines 41–49):

The overly detailed ANR background has been condensed to improve clarity while retaining key information.

The original text “Assisted Natural Regeneration (ANR) is a forest management…ANR and traditional afforestation practices.”has been revised to “[Assisted natural regeneration (ANR) is a low-cost forest restoration method that minimizes disturbance by retaining logging residues and promoting natural regeneration[5]. It has been widely used in southeastern China, West Africa, and Latin America due to its effectiveness in enhancing ecosystem services, including soil carbon sequestration, biodiversity conservation, hydrological regulation, and the enhancement of soil nutrient retention and stability[9-11]. Despite growing interest in ANR, the underlying mechanisms by which it influences SOC accumulation and stabilization, especially through interactions between microbial communities and soil properties, remain poorly understood.]”

（5）Enhanced theoretical foundation:

Additional literature and theoretical linkages have been incorporated to better support the hypotheses.

The original text “As an important nodes in the global … for 8-15% of anthropogenic emissions.”has been revised to “[Soil organic carbon (SOC) represents a major component of terrestrial carbon storage and plays a critical role in global carbon cycling[1]. Forest ecosystems, particularly in subtropical regions, are important carbon reservoirs and have been widely recognized as dynamic systems where SOC turnover is strongly influenced by vegetation type, litter input, and soil microbial processes[2-4]. Forest management, through altering above- and belowground inputs, can significantly affect soil biogeochemistry and microbial-mediated carbon dynamics[5-6]. Traditionally, monoculture plantations have been implemented across subtropical China for timber production. However, such practices have been associated with biodiversity loss, soil degradation, and SOC depletion[7-8].]”

3) **L120-128, were the treatments randomly assigned?**

Thank you for pointing out the need to clarify how the treatments were assigned. We confirm that our experimental design followed a randomized block approach. Specifically, we established 12 standard plots (20 m × 20 m) in areas with similar slope orientation and gradient, and then randomly assigned one of four stand types—natural secondary forest(SF), ANR forest dominated by *Castanopsis* *carlesii* (ANR), *Castanopsis* *carlesii* plantation (CCP), and *Cunninghamia lanceolata* plantation (CLP)—to each plot, with three replicates per treatment. We have revised the corresponding section (L120–128 in our original manuscript) to explicitly describe this randomized allocation of treatments.

**L190-191, one-way ANOVA is typically used to test the significance of treatment effects on response variables or their difference, not their relationships. please rephrase or clarify the intended purpose of this analysis.**

Thank you for pointing out the need to clarify the purpose of the one-way ANOVA. We agree that one-way ANOVA is generally used to test for differences among treatment groups rather than to analyze relationships. We have revised the relevant section to emphasize that we used one-way ANOVA specifically to determine whether SOC composition and microbial community variables differed significantly among the treatments. The revised text now states that we employed one-way ANOVA to assess treatment effects on these variables, rather than their inter-relationships.

**4) the REUSLTS section needs reorganization with more concise and accurate statements focusing on the key findings.**

**L245-261, the influencing factors (soil physicochemical properties and SMC groups) are insufficient. for example, PH should be included as it plays a critical role in microbial processes and SOC fraction dynamics. Litter and root characteristics, as major sources of SOC and microbes (particularly LC), should be provide and analyze. These are often considered essential when analyzing SOC fractions in forests. In my opinion, physicochemical properties should also be tested across treatments (as with SMC groups), and the significant variables would be considered for further analysis.**

We sincerely appreciate the reviewer’s constructive suggestions. We fully agree that expanding the analysis of key drivers (e.g., pH, litter, and roots) and reorganizing the Results section would significantly enhance the clarity and scientific rigor of our findings. The following modifications have been made to address these concerns:

Inclusion of Critical Variables (pH, Litter Storage, Fine Root Biomass). A new subsection has been added to systematically compare key properties (pH, C:N , NH₄⁺-N, NO₃⁻-N, litter, roots) across treatments.

The Results section has been restructured to prioritize key findings and logical flow，Focus on core mechanisms，The revised Sections now explicitly link SOC fractions (SOC, LC, RC) to their primary drivers (microbial activity, pH, litter/root inputs), reducing redundancy in earlier. New Figures 3 and 4 summarize the interplay between SOC fractions, microbial groups, and physicochemical drivers.

We believe these revisions comprehensively address the reviewer’s concerns by strengthening the analytical depth, clarifying causal relationships, and aligning the narrative with forest SOC dynamics literature. Thank you for the insightful feedback that has markedly improved this work.

**5) there are so many spelling, grammar and writing errors that need significant improvement. please ensure a thorough review before submission. For example, L13-14, 18-19, 25-26,,,L49, the abbreviation of SOC should be labeled when it first mentioned. And also, L57 (CNP), L139 (carbon nitrogen phosphorus).**

We deeply appreciate the reviewer's meticulous review of the manuscript's language quality. We have implemented comprehensive improvements to address these issues through the following measures.

We hope these revisions meet Reviewer 1’s expectations. Thank you for helping us enhance the manuscript’s clarity.

**Response to Reviewer2:**

**1). Title: The title appears overly broad given the scope of the study. A single study may not sufficiently support the generalization implied in the title. Consider narrowing the title to reflect the specific findings, such as emphasizing the study’s focus on carbon storage and microbial community dynamics in a particular region and forest type.**

Thank you for your valuable feedback concerning the title. We acknowledge that our study primarily focuses on carbon fractions rather than overall carbon storage. Accordingly, we have removed references to “carbon storage” in the title and replaced them with “carbon fractions.” We have also narrowed the scope to emphasize our specific findings on soil carbon fractions and microbial communities in subtropical China, as you suggested.

The revised title is:“Differences in Soil Carbon Fractions and Microbial Communities and Their Underlying Mechanisms between Assisted Natural Regeneration and Plantation Forests in Subtropical China.”

**2) Abstract: The abstract seems to be a repetition of the results and lacks key supporting data.**

Thank you for highlighting this important issue. We fully agree that the original abstract lacked sufficient supporting data and appeared repetitive. To address this, we have thoroughly restructured the abstract, adding key quantitative results and highlighting specific data points to support our primary conclusions clearly. This revision not only reduces redundancy but also provides readers with a concise yet informative summary, enhancing the abstract’s precision and comprehensiveness. We believe these improvements now effectively convey the core findings and the significance of our study.

**Line 12: Rephrase "how it performs than" to "how it performs relative to" for improved fluency and precision.**

Thank you for this helpful suggestion. During our revision, we carefully reviewed and improved the manuscript, paying particular attention to linguistic accuracy, ensuring that terminology aligns with current soil and microbial ecology standards, and refining syntax to enhance readability and academic rigor. Specifically, as suggested, we rephrased "how it performs than" to "how it performs relative to." Additionally, we consulted professional language experts and utilized specialized editing software to further ensure the overall quality of language and expression in our manuscript.

**Line 17: Clearly define "LC1" and "LC2" as "labile carbon fractions with varying turnover times" to enhance precision and accessibility for a multidisciplinary audience.**

Thank you for pointing out the ambiguity regarding LC1 and LC2. Upon careful reconsideration of our study's central focus—specifically, the dynamics of SOC fractions (LC and RC)—we have revised the manuscript to streamline and clarify our terminology. Consequently, we no longer differentiate LC into LC1 and LC2. Instead, we now consistently describe LC as the "labile carbon fraction," clearly defined based on its relatively short turnover time and sensitivity to forest management practices. These revisions enhance both precision and accessibility for multidisciplinary readers.

**Introduction: Provide a more nuanced discussion of the subtropical region’s unique climatic and edaphic conditions, emphasizing their relevance to soil carbon storage and forest restoration dynamics.**

Thank you very much for your constructive comments regarding the introduction of our manuscript. We appreciate your suggestion to provide a more nuanced discussion on the subtropical region's unique climatic and edaphic conditions and their implications for soil carbon storage and forest restoration dynamics. Accordingly, we have enhanced our introduction by incorporating a detailed description of the distinctive climate (e.g., concentrated rainfall, warm and humid conditions) and highly weathered acidic soils characteristic of subtropical regions. We have also highlighted how these features lead to low soil nutrient availability and increased susceptibility to soil erosion. Furthermore, we emphasized how frequent conversions between secondary forests and plantations, combined with intensive anthropogenic disturbances in plantation management, significantly impact soil carbon storage capacity and stability. These revisions clarify the relevance of the subtropical region to our study and underscore its suitability as an ideal region for examining the effects of forest restoration and management practices on soil carbon dynamics.

We believe these adjustments address your concerns effectively, making our introduction clearer and more comprehensive. Thank you once again for your valuable feedback.

**Line 48: Elaborate on the phrase "extensive ecosystem service functions" by detailing specific services linked to ANR, such as carbon sequestration, hydrological regulation, and biodiversity enhancement.**

Thank you very much for this helpful suggestion. As recommended, we have elaborated on the phrase "extensive ecosystem service functions" by clearly specifying the ecosystem services linked to Assisted Natural Regeneration (ANR), including soil carbon sequestration, biodiversity conservation, hydrological regulation, and the enhancement of soil nutrient retention and stability. This revision provides more detail on how ANR contributes to ecosystem functioning, thereby strengthening the clarity and comprehensiveness of our manuscript.

**Lines 118-125: This sentence is too complicated, please simplify it. In addition, please specify whether the plots were randomized or stratified based on biophysical variables like slope, aspect, and vegetation type, as these can significantly influence soil and microbial metrics.**

Thank you for pointing this out. To clarify, we have simplified the original description and explicitly emphasized that treatments were randomly assigned. Specifically, we established twelve standard plots (20 m × 20 m) with similar slope orientation and gradient in an area initially covered by uniform secondary forests. After clear-cutting, each plot was randomly assigned one of four stand types: natural secondary forest(SF), ANR forest dominated by *Castanopsis* *carlesii* (ANR), *Castanopsis* *carlesii* plantation (CCP), and *Cunninghamia lanceolata* plantation (CLP), each treatment having three replicate plots. We have revised the corresponding section (Lines 118–125 in the original manuscript) to clearly reflect this randomized experimental design, thereby addressing your concern regarding the potential influences of slope orientation and gradient on soil and microbial metrics.

**Line 135: Justify the selection of a 0-10 cm soil sampling depth by citing studies highlighting its sensitivity to anthropogenic and natural disturbances.**

Thanks for this suggestion. We agree that clarifying the rationale for sampling the 0–10 cm soil layer is important. The topsoil layer (0–10 cm) was chosen because it is most sensitive to forest management-induced changes, particularly concerning microbial community composition, soil physicochemical properties, and organic carbon dynamics. Previous studies have demonstrated that forest management practices primarily impact the surface soil, with the top 10 cm layer exhibiting significant variations in microbial biomass, community structure, and nutrient cycling, which directly affect soil carbon storage and stability (Zhao et al., 2022). Furthermore, surface soils store the majority of the total soil organic carbon pool, playing a critical role in overall soil carbon sequestration capacity (Yang et al., 2019). Therefore, the selection of 0–10 cm soil depth in this study is justified by its sensitivity and critical importance in reflecting forest management-induced changes.

**Line 144: Provide a detailed rationale for adopting the acid hydrolysis method and discuss its limitations, particularly in differentiating labile from recalcitrant carbon fractions.**

Thank you for highlighting the need for clarification regarding our method selection. We have expanded the methodology section by providing a detailed rationale for adopting the acid hydrolysis method and addressing its limitations. The acid hydrolysis method was chosen due to its efficiency, reproducibility, and widespread use in differentiating soil organic carbon (SOC) into labile carbon (LC) and recalcitrant carbon (RC) fractions. This method separates LC, which includes carbohydrates, proteins, and other easily decomposed compounds, from RC that consists mainly of chemically stable substances such as lignin, humic acids, and fulvic acids (Rovira and Vallejo, 2002). However, we recognize its limitations, including potential partial overlap between labile and recalcitrant pools due to varying resistance of soil organic matter compounds to acid hydrolysis, which may affect the precise categorization of certain intermediate carbon fractions. Despite this limitation, acid hydrolysis remains a practical and effective method to comparatively assess changes in SOC fractions in response to forest management practices, providing meaningful insights into the dynamics of carbon storage and stability.

**Figure 2: Standardize the presentation of Figure 2 by including absolute values, relative percentages, and error bars to improve interpretability. Also, please give a distinction to the subplots.**

Thank you for your helpful suggestion regarding the clarity and interpretability of Figure 2. In response, we have revised Figure 2 as follows:

Error bars (± standard error) have been added to all bars to reflect data variability;Y-axis units have been standardized to display absolute values (e.g., g·kg⁻¹ for carbon content);Subplots have been clearly distinguished with labels (SOC), (RC), and (LC), respectively. Each subplot is now referenced in the figure legend;Relative percentage changes across forest types have been described in the Results section (Lines XX–XX), to avoid overloading the figure itself.

We hope these revisions have improved the readability and comparability of the figure. Thank you for this valuable suggestion.

**Figure 4: I am confused about the graph on the right side of Figure 4. How was the data obtained (is it correct)? What does the effect here refer to? Please clarify. Moreover, please expand abbreviations like "Envi" (environmental variables) and "Mic" (microbial indicators) to facilitate interpretation by diverse readerships.**

Thank you for pointing out the unclear aspects regarding Figure 4. We acknowledge that our previous presentation might have led to confusion regarding the method and interpretation of the results. In our revised manuscript, we have clarified the analytical approach by reconstructing the structural equation model (SEM) using the R package lavaan (v0.6-16) with maximum likelihood estimation. Specifically, additional indicators such as soil pH, litter stock, and fine root biomass were incorporated into our SEM analysis, optimizing the model's explanatory power and robustness.

The "effects" in the graph now explicitly represent standardized path coefficients, indicating the strength and direction of relationships between latent and observed variables. Moreover, we have fully expanded previously ambiguous abbreviations: "Envi" is now clearly indicated as "environmental variables," and "Mic" is specified as "microbial indicators," making the figure more accessible for diverse readerships.

We have updated the Results and Discussion sections accordingly to reflect these methodological clarifications and enhanced interpretations. We believe these modifications have substantially improved the clarity and rigor of Figure 4 and associated analyses.

**Table 2: Specify the replication number (e.g., n=3) and statistical measures used to ensure transparency in data interpretation.**

Thank you very much for your suggestion. As recommended, we have revised Table by explicitly specifying the replication number (n=3) and clearly indicating the statistical measure used (mean ± standard error). These adjustments have enhanced transparency in data presentation and interpretation.

**3) Discussion: The discussion is the weakest part of this manuscript. A well-structured discussion should be based on this study's findings and combined with previous studies to discuss something new rather than simply listing and piling up the results.**

We appreciate the reviewer’s constructive feedback regarding the Discussion section. In response, we have undertaken a substantial revision of this section to improve its logical structure, depth of interpretation, and integration with existing literature.

Specifically, the revised Discussion is now organized into five thematically structured subsections:

Forest management effects on soil properties and microbial traits – this part synthesizes key findings on C/N, ammonium-N, and microbial biomass responses, and links them to shifts in litter input and decomposition processes, supported by both our data and previous studies.

Microbial responses and ecological strategies – we interpret the changes in microbial indicators (e.g., F:B, GP:GN) under different management regimes as adaptive shifts in resource use strategies, citing supporting literature .

Divergent responses of SOC fractions – we compare LC and RC losses across forest types and explain the patterns based on litter quality, decomposition stages, and substrate availability.

Biotic and abiotic regulatory pathways – drawing on the structural equation modeling (SEM) results, we discuss the dual-pathway regulation of SOC through microbial and soil physicochemical variables, highlighting the central role of LC as a mechanistic bridge between inputs and SOC accumulation.

Management implications – we conclude with insights into the implications of ANR for sustainable forest carbon management, supported by both our findings and broader ecological theory.

Throughout the revised Discussion, we focused on interpretation rather than repetition. We clarified how our findings confirm, extend, or contrast with existing studies, and emphasized the novel contribution of identifying labile carbon (LC) as a critical intermediate in SOC stabilization under humid subtropical conditions.

We hope these substantial revisions fully address the reviewer’s concern and strengthen the scientific value of our manuscript.

**4) References: Ensure full compliance with journal-specific formatting requirements, standardizing Latin names, capitalization, and citation styles.**

Thank you for highlighting this issue. We have carefully rechecked and revised the References section to ensure full compliance with the journal's specific formatting guidelines. Specifically, we have standardized Latin species names, capitalization, and citation styles throughout the manuscript to meet the publication requirements.

**5) General: please conduct a meticulous review for linguistic precision, ensure that terminology aligns with current soil and microbial ecology standards, and refine syntax for enhanced readability and academic rigor.**

Thank you for your valuable suggestion. We have meticulously reviewed and refined the manuscript for linguistic precision, ensured that all terminologies align with current standards in soil and microbial ecology, and enhanced syntax for readability and academic rigor. Additionally, we have consulted with experienced colleagues in the field and employed professional editing software to further ensure the manuscript meets high academic standards. We appreciate your guidance, which has greatly improved our manuscript's quality and clarity.

We hope these revisions meet Reviewer 2’s expectations. Thank you for helping us enhance the manuscript’s clarity.
