# Supplementary material for: Differences in soil carbon fractions and microbial communities and their underlying mechanisms between assisted natural regeneration and plantation forests in subtropical China
Source: PLoS One. 2026 Jan 8;21(1):e0339350. doi: 10.1371/journal.pone.0339350 (PMC12782447; doi:10.1371/journal.pone.0339350)
Supplement: Attachment — Submitted filename: Review PONE-D-24-53500R1.docx [file pone.0339350.s002.docx]

**Revision no. PONE-D-24-53500R1**

**“Differences in Soil Carbon Fractions and Microbial Communities and Their Underlying Mechanisms between Assisted Natural Regeneration and Plantation Forests in Subtropical China”**

Since **Table 1** reflects basic and important information of the standing trees characteristics in different stands. Therefore, I strongly suggest the authors to provide justifications on

1. How ANR forests outperform plantations in terms of belowground input and in comparison, with CCP and CLP.
2. The authors must provide a brief justification in relation with the understory biomass diversity which would enable to understand by the audiences.
3. This justification much be reflected in abstract, as it bears a meaningful insight.

This manuscript is good and covers many aspects of soil. However, I strongly agreed with the reviewer’s viewpoint which lack of sincere justification in the discussion. The revised manuscript provided shows progress.

I hope, a brief improvement of the above given problems could be a well written manuscript.
