# Supplementary material for: Differences in soil carbon fractions and microbial communities and their underlying mechanisms between assisted natural regeneration and plantation forests in subtropical China
Source: PLoS One. 2026 Jan 8;21(1):e0339350. doi: 10.1371/journal.pone.0339350 (PMC12782447; doi:10.1371/journal.pone.0339350)
Supplement: Attachment — Submitted filename: Suggestions for Improvement- PlosOne - 10-09-2025.docx [file pone.0339350.s003.docx]

**Differences in Soil Carbon Fractions and Microbial Communities and Their Underlying Mechanisms between Assisted Natural Regeneration and Plantation Forests in Subtropical China**

**Suggestions for Improvement:**

1. **ABSTRACT:**

- Replace phrases like *“remain insufficiently understood”* with simpler wording such as *“are not yet fully understood”*; also break long sentences (e.g., line 24–25) for readability.
- Use abbreviations consistently (e.g., SF) or omit them; ensure species names (*Castanopsis carlesii*, *Cunninghamia lanceolata*) are italicized throughout.
- Shorten phrases like *“SEM analysis revealed”* to *“SEM identified”*; refine awkward wording such as *“no direct influence path”* → *“no direct pathway of influence”*.
- Replace vague terms like *“SOC levels”* with more specific wording (e.g., *“SOC concentrations”*).
- The concluding sentence (lines 25–27) reads like part of the discussion; consider moving it to strengthen the conclusion section.

2. **INTRODUCTION:**

- Some sentences are overly long and could be split for readability (e.g., lines 41–43 and 53–56). Shorter sentences would help highlight key findings more clearly.
- Use abbreviations consistently: SOC, LC, RC, and ANR are well defined, but “secondary natural forest” (line 86) is not abbreviated, unlike elsewhere. Decide whether to introduce abbreviations for all forest types or avoid them.
- Replace repetitive structures such as “remain poorly understood” (line 46) and “remain inadequately understood” (line 75) with varied phrasing to improve flow.
- Line 55: “facilitate improved resources and environmental management” is vague. Consider specifying “facilitate improved soil resource use and ecosystem management”.

3. **MATERIALS & METHODS:**

- Breaking the longer sentences into shorter steps or using bullet-style formatting for better readability (e.g., lines 150–166, acid hydrolysis method).
- Abbreviations like SF, ANR, CCP, and CLP should be used consistently after introduction.
- In soil and PLFA methods, some units and symbols (e.g., g·cm⁻³, Mg·hm²) could be standardized to SI format for consistency. Check spacing in chemical formulas (e.g., H₂SO₄, NH₄⁺-N).
- Ensure uniform citation of figures and tables (e.g., Fig. 1c vs. Fig 1c; Table 1 is referenced, but caption and format could be improved for clarity and readability).
- Phrasing like “Previous studies have shown…” (line 135) or “In the present study…” (line 149) is slightly repetitive. Consider more varied transitions to improve flow.

4. **RESULTS:**

- **Clarity of Data Presentation:** Some descriptions of results (lines 218–225) are overly dense with numbers and percentages. Breaking these into shorter sentences or using parentheses sparingly would improve readability.
- **Terminology Consistency:** Ensure abbreviations (e.g., NH₄⁺-N, NO₃⁻-N, FRB, LS) are consistently defined once and used uniformly across text, tables, and figures.
- **Interpretation Balance:** The discussion of plantations emphasizes nutrient and SOC declines but offers little context about variability or possible ecological explanations (e.g., species-specific traits of *Castanopsis* vs. *Cunninghamia*). Adding one line of interpretation would strengthen this.
- **Figure/Table Referencing:** References to Table 2 and Figure 2 are appropriate, but Figure 3 and Figure 4 interpretations (lines 269–303) could briefly emphasize the ecological meaning of the significant pathways rather than focusing mainly on R² values.

5. **DISSCUSION:**

- The model fit indices (χ², df, RMSEA, CFI, TLI) are reported, but the narrative doesn’t fully explain their ecological significance. Readers unfamiliar with SEM may find it overly technical.
- The explanation of LC and RC dynamics is repeated across multiple paragraphs. This could be condensed to avoid redundancy and to keep focus on novel insights.

6. **CONCLUSION:**
   - The paragraph is breaking it into two shorter paragraphs for better readability and flow.
   - Acronyms such as **LC, RC, SOC, ANR, GP:GN** are frequently used — ensure they are defined at first use within this section for clarity to readers unfamiliar with earlier parts.
   - The use of percentages (e.g., “SOC reduced by up to 30.4%”) would benefit from specifying whether this was relative to secondary forests, ANR, or another reference system.
7. **SUGGESTIVE NOTE:**

I find this manuscript has;

- **Scientific Contribution** – The focus on SOC fractions (labile vs. recalcitrant) and their microbial underpinnings in subtropical forests is timely and significant. The finding that LC serves as a critical mediator of SOC stability provides fresh insights.
- **Methodological Rigor** – Use of multi-parameter soil and microbial analyses alongside SEM enhances the robustness of causal inference.
- **Novel Insights** – The comparison of ANR and plantations highlights both ecological and management implications, especially regarding LC-to-RC conversion pathways.
- **Data Presentation** – Tables and figures are detailed and well-organized, allowing clear visualization of results.
- **Policy Relevance** – The work provides applicable recommendations for sustainable forestry and carbon management.

The manuscript makes a valuable contribution to forest ecology, carbon cycle research, and sustainable land management. I recommend **minor revisions** focusing on clarity, consistency, and contextual framing. Once these improvements are incorporated, the manuscript will be well-prepared for acceptance.
