# Supplementary material for: Differences in soil carbon fractions and microbial communities and their underlying mechanisms between assisted natural regeneration and plantation forests in subtropical China
Source: PLoS One. 2026 Jan 8;21(1):e0339350. doi: 10.1371/journal.pone.0339350 (PMC12782447; doi:10.1371/journal.pone.0339350)
Supplement: Attachment — Submitted filename: Response to Reviewers.docx [file pone.0339350.s005.docx]

**Response to Reviewer2:**

**1) The authors have addressed the comments effectively; however, there are still some spelling, formatting, and grammatical errors present. For instance, abbreviations are repeated unnecessarily, and variables are not italicized as required. Specific examples include lines 39, 90, 92, 93, 139, 208, 209, and 214. A thorough review of the manuscript is needed to correct these issues.**

Thank you once again for reviewing our manuscript and pointing out these details that require improvement. We sincerely apologize for the spelling, formatting, and grammatical errors that remained in the manuscript. Such oversights should not have occurred.

Based on your valuable feedback, we have conducted a thorough, word-by-word review of the entire manuscript (not limited to the specific lines mentioned) to correct all spelling and grammatical errors. We have also eliminated all unnecessary repetitions of abbreviations, ensuring that each abbreviation is defined upon its first occurrence and used consistently thereafter. The issues in the lines you specifically pointed out (39, 90, 92, 93, 139, 208, 209, and 214) have been fully addressed.

We believe that this revision has significantly improved the linguistic quality and formatting consistency of the manuscript. Once again, we greatly appreciate the time and effort you have dedicated to providing us with such rigorous and constructive feedback, which has been essential in refining our work.

**Response to Reviewer 4:**

**1). How ANR forests outperform plantations in terms of belowground input and in comparison, with CCP and CLP.**

**Response:**
We added plot-level evidence from **Table 1** showing that **fine-root yield** is much higher under ANR (**284 g m⁻² yr⁻¹**) than in **CCP (163)** and **CLP (145)**. We explain that higher belowground inputs increase substrate supply to microbes, supporting higher microbial biomass and **LC** retention, which in turn promotes **SOC** accumulation.

**Changes in manuscript (key sentence added to Discussion – first subsection)**
“**Direct plot measurements (Table 1) support stronger belowground inputs under ANR: fine-root yield is 284 g m⁻² yr⁻¹ in ANR vs 163 in CCP and 145 in CLP, which likely enhances substrate supply to microbes and helps sustain LC pools and microbial biomass relative to plantations.**”

**2) The authors must provide a brief justification in relation with the understory biomass diversity which would enable to understand by the audiences.**

**Response:**

We added **understory cover** data from **Table 1** (ANR 92.7%, comparable to SF ≈ 93.3%, vs. CCP ≈ 84.5% and CLP 82.1%) as a field-based proxy for a richer, more functionally diverse understory. We clarify how this richer understory increases litter and rhizodeposition, thereby supporting microbial activity and **LC** retention.

**Changes in manuscript (key sentence added to Discussion – first subsection)**
“**Understory cover in ANR (92.7%) is comparable to SF ( 93.3%) but exceeds CCP (84.5%) and CLP (82.1%), indicating a richer understory that provides additional litter and root inputs to sustain microbial activity and LC.**”

**3) This justification much be reflected in abstract, as it bears a meaningful insight.**

**Response:**

We revised the **Abstract** to succinctly reflect ANR’s advantage in belowground inputs and understory structure, while keeping the abstract concise and mechanism-focused.

**Changes in manuscript (one-sentence addition to Abstract)**
“**At our site, ANR also exhibited higher fine-root input and richer understory cover than plantations, supporting microbially mediated LC retention and, ultimately, greater SOC stabilization.**”

**Response to Reviewer 5:**

1. **ABSTRACT:**

- **Replace phrases like *“remain insufficiently understood”* with simpler wording such as *“are not yet fully understood”*;**

**Response:**

Done. We simplified the phrasing to improve readability while preserving the original meaning.

**Changes in manuscript:**

Original: “...influence soil organic carbon (SOC) stability **remain insufficiently understood**.”
Revised: “...influence soil organic carbon (SOC) stability **are not yet fully understood**.”

- **also break long sentences (e.g., line 24–25) for readability.**

**Response:**

Done. We split the long sentence describing the two SEM pathways into shorter sentences and also separated the sentence about recalcitrant carbon into two sentences for clarity.

**Changes in manuscript (key excerpts):**

“**SEM identified** two partially independent regulatory pathways. **In the first pathway, aboveground litter inputs altered soil pH, carbon:nitrogen ratio, and ammonium nitrogen. These changes promoted the conversion of labile carbon into recalcitrant carbon and increased total SOC. In the second pathway,** fine root biomass enhanced labile carbon accumulation by increasing the abundance of Gram-positive bacteria.”

“**Recalcitrant carbon is conventionally considered a stable SOC component. However, the SEM identified no direct pathway of influence** from recalcitrant carbon to total SOC.”

- Use abbreviations consistently (e.g., SF) or omit them; ensure species names (*Castanopsis carlesii*, *Cunninghamia lanceolata*) are italicized throughout.

**Response:**

Done. Because “secondary forest (SF)” is not reused in the abstract, we removed the unused abbreviation and kept “secondary forest” in full. We retained ANR, which is used multiple times. We also ensured that species names are italicized.

**Changes in manuscript (key excerpts):**

“...four forest management systems in subtropical China: **a secondary forest, an assisted natural regeneration (ANR) forest, and two monoculture plantations dominated by *Castanopsis carlesii* and *Cunninghamia lanceolata***.”

- Shorten phrases like *“SEM analysis revealed”* to *“SEM identified”*; refine awkward wording such as *“no direct influence path”* → *“no direct pathway of influence”*.

**Response:**

Done. We revised both phrases as suggested.

**Changes in manuscript (key excerpts):**

 “**SEM identified** two partially independent regulatory pathways...”

 “...**no direct pathway of influence** from recalcitrant carbon to total SOC.”

- Replace vague terms like *“SOC levels”* with more specific wording (e.g., *“SOC concentrations”*).

**Response:**

Done. We now use “SOC concentrations” in the results statement.

**Changes in manuscript (key excerpts):**

Original: “ANR maintained SOC **levels** and microbial traits...”
Revised: “ANR maintained **SOC concentrations** and microbial characteristics...”

- The concluding sentence (lines 25–27) reads like part of the discussion; consider moving it to strengthen the conclusion section.

**Response:**

We streamlined the final statements to focus on the key findings and their immediate implications, removing the discussion-like tone. The abstract now ends with a concise, results-grounded implication for management. We will also ensure that the Conclusion section elaborates on these implications to avoid redundancy.

**Changes in manuscript (key excerpts):**

“**Overall, our findings demonstrate that ANR mitigates soil carbon losses by enhancing microbially mediated carbon retention and reducing disturbance. This approach preserves SOC through improved litter dynamics and root–microbe interactions, providing insights for sustainable forest carbon management.**”

2. **INTRODUCTION:**

- Some sentences are overly long and could be split for readability (e.g., lines 41–43 and 53–56). Shorter sentences would help highlight key findings more clearly.
- Use abbreviations consistently: SOC, LC, RC, and ANR are well defined, but “secondary natural forest” (line 86) is not abbreviated, unlike elsewhere. Decide whether to introduce abbreviations for all forest types or avoid them.
- Replace repetitive structures such as *“remain poorly understood”* (line 46) and *“remain inadequately understood”* (line 75) with varied phrasing to improve flow.
- Line 55: *“facilitate improved resources and environmental management”* is vague. Consider specifying *“facilitate improved soil resource use and ecosystem management”*.

**Response:**

We thank the reviewer for the helpful suggestions on the Introduction. In the revised manuscript we improved readability and consistency without altering the scientific content. First, we split overly long sentences—including those flagged previously at lines 41–43 and 53–56—into shorter, focused statements (e.g., the sentences describing ANR’s benefits and the subtropical soil–climate context are now presented as two to three concise sentences each). Second, we adopted a consistent abbreviation scheme for all forest types to enhance clarity throughout the paper: secondary forest (SF), assisted natural regeneration (ANR), Castanopsis carlesii plantation (CCP), and Cunninghamia lanceolata plantation (CLP), each introduced at first mention and used thereafter to align with the Methods/Results sections. Third, to avoid repetitive constructions, we replaced phrases such as “remain poorly understood” and “remain inadequately understood” with varied and precise alternatives (e.g., “are not well characterized,” “are still unclear,” and “remains underexplored”) where appropriate. Fourth, we clarified vague wording by revising “facilitate improved resources and environmental management” to the more specific “facilitate improved soil resource use and ecosystem management.” We also made minor style and terminology refinements (e.g., “microbial-mediated” → “microbially mediated,” active-voice revisions, and consistent italicization of species names). These edits sharpen the framing of our research questions and highlight the study’s contributions more clearly.

3. **MATERIALS & METHODS:**

- Breaking the longer sentences into shorter steps or using bullet-style formatting for better readability (e.g., lines 150–166, acid hydrolysis method).
- Abbreviations like SF, ANR, CCP, and CLP should be used consistently after introduction.
- In soil and PLFA methods, some units and symbols (e.g., *g·cm⁻³*, *Mg·hm²*) could be standardized to SI format for consistency. Check spacing in chemical formulas (e.g., *H₂SO₄*, *NH₄⁺-N*).
- Ensure uniform citation of figures and tables (e.g., *Fig. 1c* vs. *Fig 1c*; *Table 1* is referenced, but caption and format could be improved for clarity and readability).
- Phrasing like *“Previous studies have shown…”* (line 135) or *“In the present study…”* (line 149) is slightly repetitive. Consider more varied transitions to improve flow.

**Response:**

We appreciate the reviewer’s suggestions and have revised the Materials & Methods to improve readability, consistency, and reproducibility without changing the underlying procedures or results. Specifically, we (i) split long sentences and rewrote the acid-hydrolysis protocol into numbered steps, clarifying volumes, temperatures, times, pore sizes, and centrifugation settings (with approximate ×g) and explicitly defining LC1, LC2, total LC, and RC; (ii) standardized abbreviations and now use SF, ANR, CCP, and CLP consistently throughout this section; (iii) harmonized units and chemical notation to SI format, including °C, mol L⁻¹, µm, Mg ha⁻¹ (converted from Mg hm⁻²), and proper ionic/chemical formatting (e.g., H₂SO₄, NH₄⁺-N, NO₃⁻-N), and corrected terms (e.g., “silt”); (iv) unified figure/table citations (e.g., “Fig. 1”, “Fig. 1c”) and streamlined the Fig. 1 caption for clarity; and (v) varied repetitive lead-ins (e.g., avoiding “Previous studies have shown…” / “In the present study…”) and aligned tense/voice, with minor editorial corrections (e.g., consistent past tense, species italicization where relevant). We believe these edits substantially enhance clarity and ease of replication while preserving the scientific content.

4. **RESULTS:**

- **Clarity of Data Presentation:** Some descriptions of results (lines 218–225) are overly dense with numbers and percentages. Breaking these into shorter sentences or using parentheses sparingly would improve readability.
- **Terminology Consistency:** Ensure abbreviations (e.g., NH₄⁺-N, NO₃⁻-N, FRB, LS) are consistently defined once and used uniformly across text, tables, and figures.
- **Interpretation Balance:** The discussion of plantations emphasizes nutrient and SOC declines but offers little context about variability or possible ecological explanations (e.g., species-specific traits of *Castanopsis* vs. *Cunninghamia*). Adding one line of interpretation would strengthen this.
- **Figure/Table Referencing:** References to Table 2 and Figure 2 are appropriate, but Figure 3 and Figure 4 interpretations (lines 269–303) could briefly emphasize the ecological meaning of the significant pathways rather than focusing mainly on R² values.

**Response:**

We thank the reviewer for the constructive comments. In the Results we (i) reduced sentence density and numbers-per-sentence by splitting long sentences and using concise parentheses where appropriate, improving readability in lines 218–225 and related passages; (ii) enforced terminology consistency, ensuring that NH₄⁺-N, NO₃⁻-N, FRB, LS, LC, RC, PLFA are defined at first mention and used uniformly across text, tables, and figures; (iii) added a brief ecological interpretation to contextualize plantation differences, noting species-specific litter quality and rooting traits of Castanopsis (CCP) versus Cunninghamia (CLP) that likely underlie contrasts in microbial biomass and SOC fractions; and (iv) refined the Fig. 3–4 narrative to emphasize the ecological meaning of the SEM pathways (litter→C:N/pH→NH₄⁺ and FRB→GP→LC) while keeping model metrics concise. These revisions enhance clarity without altering the reported statistics or conclusions.

5. **DISSCUSION:**

- The model fit indices (χ², df, RMSEA, CFI, TLI) are reported, but the narrative doesn’t fully explain their ecological significance. Readers unfamiliar with SEM may find it overly technical.

**Response:**
Thank you. We revised the **Discussion** to provide a plain-language, ecological interpretation of the indices and limited reporting to the indices actually used in our analyses (**χ² = 35.91, df = 24, P = 0.056; RMSEA = 0.203**). We briefly explain what these values imply for ecological inference and why we emphasize the **direction and magnitude of standardized paths** when interpreting mechanisms.

**Changes in manuscript (key sentences added):**

Discussion (Influence Pathways of Forest Management on SOC Pool):

“**The overall fit statistics (χ² = 35.91, df = 24, P = 0.056; RMSEA = 0.203) indicate marginal global misfit but an informative structure. Ecologically, this means the hypothesized pathways capture the dominant processes—(i) greater litter inputs elevating C:N and lowering pH, which increase NH₄⁺-N and promote LC→RC conversion and higher SOC; and (ii) larger FRB increasing GP abundance and boosting LC—while the elevated RMSEA cautions that additional, unmeasured processes (e.g., microclimate or substrate chemistry) may also contribute given n = 12.**”

- The explanation of LC and RC dynamics is repeated across multiple paragraphs. This could be condensed to avoid redundancy and to keep focus on novel insights.

**Response:**

We agree and condensed the LC/RC narrative by removing repetitive background sentences and merging overlapping explanations into concise mechanism-focused statements. The revision keeps the emphasis on what is novel (i.e., the dual pathways and the central role of LC in humid subtropics) while retaining essential references.

Changes in manuscript (before → after examples)

 *Removed repetitive background* (deleted):
“”

 *Replaced with concise mechanism statement* (added):
“**Because LC derives from easily decomposable substrates and is disturbance-sensitive, continuous litterfall and root exudates in SF/ANR likely sustain LC pools, whereas early site preparation and understory removal in plantations reduce inputs and deplete LC.**”

 *RC paragraph tightened* (kept results; trimmed explanation):
“**RC, composed of chemically complex residues, underpins long-term SOC stabilization; higher RC in SF/ANR likely reflects reduced disturbance and long-term litter inputs, whereas the lowest RC in CLP is consistent with slower litter decomposition and nutrient return.**”

6. **CONCLUSION:**
   - The paragraph is breaking it into two shorter paragraphs for better readability and flow.
   - Acronyms such as **LC, RC, SOC, ANR, GP:GN** are frequently used — ensure they are defined at first use within this section for clarity to readers unfamiliar with earlier parts.
   - The use of percentages (e.g., *“SOC reduced by up to 30.4%”*) would benefit from specifying whether this was relative to secondary forests, ANR, or another reference system.

**Response:**

Thank you for the helpful suggestions. We have revised the Conclusions as follows to improve clarity and readability without changing the scientific content:

1. **Paragraphing:** The original single paragraph has been **split into two shorter paragraphs** to improve flow.
2. **Acronyms defined at first use within this section:** We now define **SOC** (soil organic carbon), **ANR** (assisted natural regeneration), **SF** (secondary forest), **LC** (labile carbon), **RC** (recalcitrant carbon), **NH₄⁺–N** (ammonium nitrogen), **GP:GN** (Gram-positive:Gram-negative), and **SEM** (structural equation modeling).
3. **Reference category for percentages clarified:** The statement on SOC decline now reads **“SOC reduced by up to 30.4% relative to SF.”**
4. **Minor wording/notation improvements:** e.g., “SOC **concentrations**,” and “**C:N** ratio.”

These edits address the reviewer’s concerns on readability, acronym clarity, and percentage references.

7. **SUGGESTIVE NOTE:**

I find this manuscript has;

- **Scientific Contribution** – The focus on SOC fractions (labile vs. recalcitrant) and their microbial underpinnings in subtropical forests is timely and significant. The finding that LC serves as a critical mediator of SOC stability provides fresh insights.
- **Methodological Rigor** – Use of multi-parameter soil and microbial analyses alongside SEM enhances the robustness of causal inference.
- **Novel Insights** – The comparison of ANR and plantations highlights both ecological and management implications, especially regarding LC-to-RC conversion pathways.
- **Data Presentation** – Tables and figures are detailed and well-organized, allowing clear visualization of results.
- **Policy Relevance** – The work provides applicable recommendations for sustainable forestry and carbon management.

The manuscript makes a valuable contribution to forest ecology, carbon cycle research, and sustainable land management. I recommend **minor revisions** focusing on clarity, consistency, and contextual framing. Once these improvements are incorporated, the manuscript will be well-prepared for acceptance.
